# Phosphorylated SARM1 is involved in the pathological process of rotenone-induced neurodegeneration

**DOI:** 10.1093/jb/mvad068

**Published:** 2023-09-19

**Authors:** Hitoshi Murata, May Tha Zin Phoo, Toshiki Ochi, Nahoko Tomonobu, Ken-ichi Yamamoto, Rie Kinoshita, Ikuko Miyazaki, Masahiro Nishibori, Masato Asanuma, Masakiyo Sakaguchi

**Affiliations:** Department of Cell Biology, Okayama University Graduate School of Medicine, Dentistry and Pharmaceutical Sciences, 2-5-1 Shikata-cho, Kita-ku, Okayama 700-8558, Japan; Department of Cell Biology, Okayama University Graduate School of Medicine, Dentistry and Pharmaceutical Sciences, 2-5-1 Shikata-cho, Kita-ku, Okayama 700-8558, Japan; Department of Cell Biology, Okayama University Graduate School of Medicine, Dentistry and Pharmaceutical Sciences, 2-5-1 Shikata-cho, Kita-ku, Okayama 700-8558, Japan; Department of Cell Biology, Okayama University Graduate School of Medicine, Dentistry and Pharmaceutical Sciences, 2-5-1 Shikata-cho, Kita-ku, Okayama 700-8558, Japan; Department of Cell Biology, Okayama University Graduate School of Medicine, Dentistry and Pharmaceutical Sciences, 2-5-1 Shikata-cho, Kita-ku, Okayama 700-8558, Japan; Department of Cell Biology, Okayama University Graduate School of Medicine, Dentistry and Pharmaceutical Sciences, 2-5-1 Shikata-cho, Kita-ku, Okayama 700-8558, Japan; Department of Medical Neurobiology, Okayama University Graduate School of Medicine, Dentistry and Pharmaceutical Sciences, 2-5-1 Shikata-cho, Kita-ku, Okayama 700-8558, Japan; Department of Translational Research and Drug Development, Okayama University Graduate School of Medicine, Dentistry and Pharmaceutical Sciences, 2-5-1 Shikata-cho, Kita-ku, Okayama 700-8558, Japan; Department of Medical Neurobiology, Okayama University Graduate School of Medicine, Dentistry and Pharmaceutical Sciences, 2-5-1 Shikata-cho, Kita-ku, Okayama 700-8558, Japan; Department of Cell Biology, Okayama University Graduate School of Medicine, Dentistry and Pharmaceutical Sciences, 2-5-1 Shikata-cho, Kita-ku, Okayama 700-8558, Japan

**Keywords:** JNK, PARK2, Parkinson’s disease, Phosphorylation, SARM1.*Abbreviations:* ARM, armadillo/HEAT motif; DMSO, dimethyl sulfoxide; EGTA, ethylene glycol-bis(2-aminoethelether)-N: N: N: N-tetraacetic acid; iPSC, induced pluripotent stem cell; JNK, c-Jun N-terminal kinase; NAD, nicotinamide adenine dinucleotide; NSC, neural stem cell; NF-L, neurofilament-L; NF-M, neurofilament-M; PD, Parkinson’s disease; PINK1, PTEN-induced kinase 1; ROS, reactive oxygen species; SAM, sterile alpha motif; SARM1, sterile alpha and Toll/interleukin receptor motif-containing protein 1; SNpc, substantia nigra pars compacta; TH, tyrosine hydroxylase; TIR, Toll/interleukin receptor; WT, wild type

## Abstract

Sterile alpha and Toll/interleukin receptor motif-containing protein 1 (SARM1) is a NAD^+^ hydrolase that plays a key role in axonal degeneration and neuronal cell death. We reported that c-Jun N-terminal kinase (JNK) activates SARM1 through phosphorylation at Ser-548. The importance of SARM1 phosphorylation in the pathological process of Parkinson’s disease (PD) has not been determined. We thus conducted the present study by using rotenone (an inducer of PD-like pathology) and neurons derived from induced pluripotent stem cells (iPSCs) from healthy donors and a patient with familial PD PARK2 (FPD2). The results showed that compared to the healthy neurons, FPD2 neurons were more vulnerable to rotenone-induced stress and had higher levels of SARM1 phosphorylation. Similar cellular events were obtained when we used PARK2-knockdown neurons derived from healthy donor iPSCs. These events in both types of PD-model neurons were suppressed in neurons treated with JNK inhibitors, Ca^2+^-signal inhibitors, or by a SARM1-knockdown procedure. The degenerative events were enhanced in neurons overexpressing wild-type SARM1 and conversely suppressed in neurons overexpressing the SARM1-S548A mutant. We also detected elevated SARM1 phosphorylation in the midbrain of PD-model mice. The results indicate that phosphorylated SARM1 plays an important role in the pathological process of rotenone-induced neurodegeneration.

## Graphical Abstract

**Graphical Abstract:**
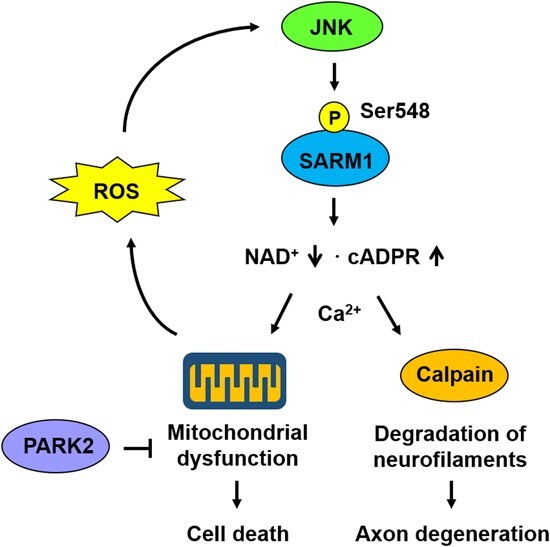

Axonal degeneration is a hallmark of several neurological disorders, including peripheral neuropathy, traumatic brain injury, and neurodegenerative diseases such as amyotrophic lateral sclerosis, Alzheimer’s disease, and Parkinson’s disease (PD) *(**1*–*3**)*. The elucidation of the mechanisms involved in axonal degeneration is important for understanding the disease onset and subsequent progression, and the clarification of the underlying mechanisms will contribute to the establishment of effective therapeutic methods targeting the above-mentioned diseases, which have been challenging to treat.

In PD, the degeneration of dopaminergic neurons that project from the substantia nigra to the striatum is the primary cause of typical symptoms of PD, i.e., the loss of proper control of body movements. Dopaminergic neurons possess widely spread and highly dense axonal arborizations in the striatum *(**4**)*. Axonal degeneration is observed at an early stage of PD and is involved in the progression of the pathogenesis *(**5**)*. However, the mechanism(s) by which axonal degeneration is triggered in PD are not fully understood. One of the potential causes is mitochondrial dysfunction. It has been suggested that mitochondrial dysfunction is involved in axonal degeneration *(**6**,**7**)*.

PINK1 (gene name, *PINK1* or *PARK6*) and PARK2 (gene name, *PARK2* or *PRKN*) are the causative gene products for juvenile PD and are molecules involved in mitophagy, a process by which defective mitochondria are removed from cells *(**8**,**9**)*. Since mutations in *PINK1* and *PARK2* genes disturb the induction of mitophagy, an accumulation of defective mitochondria occurs in neuronal cells. The defective mitochondria are harmful to cells because they actively produce reactive oxygen species (ROS). It has therefore been speculated that the intracellularly elevated level of ROS from the dysfunctional mitochondria induces axonal degeneration and neuronal cell death, which in turn are associated with the progression of PD. In fact, mitochondrial dysfunction triggered by *PINK1*-deletion mutation has been reported to cause axonal degeneration *(**10**)*, suggesting that PINK1 and its relevant molecules play important roles in the PD process towards its progression.

Sterile alpha and Toll/interleukin receptor motif containing protein 1 (SARM1) is a nicotinamide adenine dinucleotide (NAD)^+^ hydrolase that plays a crucial role in axonal degeneration and neuronal cell death by regulating NAD^+^ metabolism *(**11*–*13**)*. Several reports suggest that SARM1 is involved in the progression of PD. For example, rotenone, a mitochondrial complex I inhibitor that induces PD-like pathology, causes a loss of dopaminergic neurons through the induction of SARM1 expression *(**14**)*. A genetic ablation of *SARM1* works against rotenone-induced neuronal cell death *(**7**)*. In addition, Peters et al. reported an interesting event in *Sarm1*-deficient mice, in which they noticed that compared to wild-type (WT) mice, the appearance of the severe degeneration of dopaminergic axons that were distant from the 6-hydroxydopamine (6-OHDA)-affected lesion of the medial forebrain bundle was significantly delayed *(**15**)*.

These regulatory functions of SARM1 in neuronal cells and axons depend on its protein conformation. SARM1 has multiple domains, including a mitochondrial-targeting signal, an armadillo/HEAT motif (ARM) domain, tandem sterile alpha motif (SAM) domains, and a Toll/interleukin-1 receptor (TIR) domain *(**16**,**17**)*. The ARM domain is a self-inhibitory domain that traps SARM1 in an inactive state *(**18**,**19**)*. The SAM domain is required for multimer formation, and the TIR domain has NAD^+^ cleavage activity. Essuman et al. demonstrated that TIR domains from many prokaryotic proteins possess intrinsic NAD^+^ cleavage activity *(**20**)*. Interestingly, plant TIR domains also possess this activity (as do animal TIR domains), and their detailed structures were recently described *(**21**,**22**)*.

On the other hand, we showed that SARM1 inhibits mitochondrial respiration and induces an accumulation of PINK1 on mitochondria, which is followed by the induction of mitophagy *(**23**)*. Mutations in the *PINK1* gene may impair the removal of abnormal mitochondria due to the degradation of NAD^+^ with SARM1’s activation. The activation machinery of SARM1 was shown to be involved in its altered conformation at the protein level by Zhao et al., who used a cell-permeant mimetic of nicotinamide mononucleotide (NMN) to monitor the structural changes of SARM1 towards its activation *(**24**)*.

We further reported that c-Jun N-terminal kinase (JNK) phosphorylates SARM1 at Ser-548 in oxidative stress conditions and that JNK regulates the NAD^+^-cleavage activity of SARM1 *(**25**)*, suggesting a close linkage between JNK-mediated SARM1 phosphorylation and the alteration of SARM1’s conformation. SARM1 thus has a role in axonal degeneration through the reduction of the NAD^+^ level, caused in part by the phosphorylation modification of SARM1, which may be associated with PD’s progression.

To investigate this point, we sought to determine whether phosphorylated SARM1 at Ser-548 has an important role in the axonal degeneration that is linked to the progression of PD. For this purpose, we used familial PD patients’ iPSCs, neurons from healthy individual, neurons with altered expression levels of SARM1, and mouse models of PD treated with rotenone.

## Materials and Methods

### Chemicals and antibodies

Rotenone and EGTA were purchased from Sigma-Aldrich (St. Louis, NO, USA). JNK inhibitors (JNK inhibitor VIII and SP600125) and a calpain inhibitor (Calpain inhibitor III) were purchased from Cayman Chemical (Ann Arbor, MI). The following antibodies were used: rabbit anti-SARM1 antibody (Cell Signaling Technologies [CST] Beverly, MA, cat# 13022, dilution 1:500 in Can Get Signal Solution 1 [TOYOBO, Osaka, Japan]), rabbit anti-cJun (CST, cat# 9165, dilution 1:500 in Can Get Signal Solution 1), rabbit anti-phospho-cJun (CST, cat# 3270, dilution 1:500 in Can Get Signal Solution 1), rabbit anti-cleaved caspase-3 [Cl. Caspase 3] (CST, cat# 9664, dilution 1:250 in Can Get Signal Solution 1), mouse anti-Neurofilament-M [NF-M] (CST, cat# 2838, dilution 1:1000 in 10% skim milk), mouse anti-Neurofilament-L [NF-L] (Santa Cruz Biotechnology, Santa Cruz, CA; cat# c-20,012, dilution 1:1000 in 10% skim milk), mouse anti-PARK2 (Santa Cruz Biotechnology, cat# sc-32,282, dilution 1:200 in Can Get Signal Solution 1), Tyrosine hydroxylase [TH] (Santa Cruz Biotechnology, cat# sc-25,269, dilution 1:1000 in 10% skim milk), and mouse anti-β-Actin (Sigma-Aldrich, cat #A2228, dilution 1:20000 in 10% skim milk).

A mouse monoclonal antibody against phospho-Ser548 SARM1 (dilution 1:500 in 3% bovine serum albumin (BSA)/TBST (Tris-buffered saline, 0.1% Tween 20) was generated by ITM Co. (Matsumoto, Japan). The mAb was produced by immunizing animals with a synthetic phospho-peptide corresponding to residues surrounding Ser548 of human SARM1 (AAREMLHpSPLPCTGG).

### Cell culture

HEK293T cells were cultured in DMEM/F12 medium (Thermo Fisher Scientific, Waltham, MA) supplemented with 10% fetal bovine serum (FBS). SNL76/7 cells were cultured in KnockOut™ DMEM (Thermo Fisher Scientific) supplemented with 10% FBS and 2 mM L-glutamine and were used as feeder cells after treatment with 12 μg/ml mitomycin C (Sigma-Aldrich) for 2.5 hr.

To obtain human matured neuronal cells in a culture system, we used induced pluripotent stem cells (iPSCs) that were differentiated according to the following established protocol. 201B7 iPSCs derived from a healthy donor (a 36-year-old female) and 585A1 iPSCs derived from a healthy donor (a male in his 30s) were purchased from RIKEN BRC (Tsukuba, Japan) as control cells. iPSCs derived from a male patient in his 50s with familial Parkinson’s disease PARK2 with a homozygous deletion of *PARK2* exons (FPD2) were kindly provided by Dr. H. Okano *(**26**)*. Control iPSCs and FPD2 iPSCs were maintained on feeder cells in DMEM/F12 medium supplemented with 20% KnockOut™ Serum Replacement (Thermo Fisher Scientific), 1% non-essential amino acid (Sigma-Aldrich), 2 mM L-glutamine, 80 μM β-mercaptoethanol and 5 ng/ml bFGF (Wako Chemicals, Osaka, Japan). All of the experimental procedures for iPSCs were approved by the Okayama University Ethics Committee.

The induction of neural stem cells (NSCs) from iPSCs was done using a PSC neural induction medium (Thermo Fisher Scientific) according to the manufacturer’s instructions. After neural induction for 7 days, P0 NSCs were expanded in neural expansion medium on a coated dish with Geltrex™ LDEV-Free hESC-qualified reduced growth factor basement membrane matrix (Thermo Fisher Scientific). To make the Geltrex-coated dish, a dish was incubated with the Geltrex matrix solution (1:100 with Neurobasal™ medium; Thermo Fisher Scientific) for 1 hr. For differentiation into neurons, NSCs were cultured at a density of 1.5 ~ 2.2 × 10^5^ cells/cm^2^ on 0.002% poly-L-lysine (Sigma-Aldrich) and 10 μg/ml of laminin (Thermo Fisher Scientific)-coated dishes in Neurobasal Plus™ medium (Thermo Fisher Scientific) supplemented with 2% B-27 Plus™ Supplement (Thermo Fisher Scientific), 2 mM GlutaMAX™ Supplement (Thermo Fisher Scientific), CultureOne™ Supplement (Thermo Fisher Scientific), and 200 μM ascorbic acid (Sigma-Aldrich). Spent media were changed every 3 days, and 0.1% dimethyl sulfoxide (DMSO) (control) or 10 μM rotenone was added at day 6 for 24 hr.

### Transfection of plasmids and the establishment of stable transformants of NSCs

Conventional molecular biological techniques were used to generate expression constructs of C-terminal Flag-tagged human wild-type SARM1, SARM1 mutant, and N-terminal HA-tagged human JNK1. A point mutation of SARM1 (S548A) and deletion mutation of SARM1 (ΔTIR, deletion of 551-724 amino acids) were generated by the inverse polymerase chain reaction (PCR) method using a KOD-Plus mutagenesis kit (Toyobo). All expression constructs were sequenced to ensure that the fusion was in the correct reading frame and there were no additional mutations.

The pSAKA-1B (1B) vector was used to obtain stable transformants of NSC clones expressing wild-type SARM1 or SARM1-S548A mutant *(**27**)*. To establish the gene-engineered NSCs that overexpress SARM1s, 201B7 NSCs were transfected with a set of plasmids, including the 1B-SARM1 (WT or S548A) plasmid, the 1B-transposase plasmid, and the 1B-puromycin resistance gene plasmid using Lipofectamine™ Stem Transfection Reagent (Thermo Fisher Scientific). The 1B-empty-gene plasmid was used instead of the 1B-SARM1 plasmid to obtain the control NSCs. After 48 hr, the transfected NSCs were treated with 0.5 μg/ml puromycin, and positive clones that stably expressed foreign SARM1 with puromycin resistance gene were selected. The selected NSC clones were differentiated into neurons by the same procedures as those described above.

For CRISPR/gRNA studies, we used a CRISPR-Cas9-mediated gene editing system. TrueGuide synthetic guide RNAs (gRNAs; Thermo Fisher Scientific) targeting PARK2 (Cat#: A35533, Target DNA sequence: AGTCTAAGCAAATCACGTGG, Target exon: exon 6) and SARM1 (Cat#: A35511, Target DNA sequence: GTGTCGCTTCTTCGCCATGT, Target exon: exon 1) were used. First, 15 pmol of guide RNAs, 15 pmol of TrueCut Cas9 protein v2 (Thermo Fisher Scientific), and Lipofectamine Stem Transfection Reagent (Thermo Fisher Scientific) were mixed and incubated in Opti-MEM™ medium (Thermo Fisher Scientific) for 10 min. 201B7 NSCs were then transfected with these mixtures, the 1B-transposase plasmid, and the 1B-puromycin resistance gene plasmid using Lipofectamine Stem Transfection Reagent. Control NSCs were only transfected with the 1B-transposase plasmid and the 1B-puromycin resistance gene plasmid. After 48 hr, the transfected NSCs were treated with 0.5 μg/ml puromycin, and the surviving cells were collected under bulk conditions. The two cell lines that had the lowest expressions of target genes were selected. These selected NSC lines were differentiated into neurons by the same procedures as those described above.

### Animals

All experimental procedures were performed in accordance with the Okayama University Advanced Science Research Center’s Guidelines for Animal Experiments and were approved by the Animal Care and Use Committee of Okayama University Advanced Science Research Center. Eight-week-old male C57BL/6 J mice were purchased from Charles River Japan (Yokohama, Japan), housed with a 12-hr light/dark cycle at a constant temperature (23 °C), and given at libitum access to food.

### Injection of rotenone into mice and preparation of brain samples

Nine-week-old male C57BL/6 J mice weighing approximately 25 g received a daily subcutaneous injection of rotenone (*n* = 3, 5, or 10 mg/kg/day, Sigma-Aldrich) for 4 weeks via an osmotic mini-pump (#2004, Alzet, Cupertino, CA). The mini-pump was filled with rotenone dissolved in a vehicle solution consisting of equal volumes of dimethyl sulfoxide (DMSO) and polyethylene glycol 400 (PEG400). Mice were anesthetized by isoflurane inhalation. Rotenone-filled pumps were implanted under the skin on the backs of the mice. Control mice received only the vehicle solution.

For western blotting using midbrain parts, mice were euthanized by isoflurane inhalation, and the brains were surgically removed in a speedy manner. The midbrain parts containing the substantia nigra pars compacta (SNpc) were homogenized and incubated with N-PER™ neuronal protein extraction reagent (Thermo Fisher Scientific) for 20 min. After centrifugation, the supernatants were mixed with sodium dodecyl sulphate (SDS) sample buffer.

For immunohistochemistry using slices of the brain, mice were perfused with saline followed by 4% paraformaldehyde (PFA) under deep isoflurane anesthesia. The perfused brains were removed immediately and post-fixed for 24 hr in 4% PFA. Following cryoprotection in 15% sucrose with phosphate buffer for 48 hr, the brains were snap-frozen with powdered dry ice, and then 20-μm-thick sections were cut on a cryostat. Brain slices were collected at levels containing the SNpc (−2.8 to −3.0 mm from bregma).

### Western blot analysis

We performed a western blot analysis under conventional conditions after lysing cells using an SDS sample buffer with PhosphoSTOP (Roche, Penzberg, Germany). Five-μg protein extracts were applied to the SDS-PAGE (polyacrylamide gel electrophoresis) and electro-transferred onto an Immobilon membrane (Millipore, Bedford, MA). To detect immunoreactive proteins, we used horseradish peroxidase (HRP)-conjugated anti-mouse or anti-rabbit secondary antibodies (Cell Signaling Technologies) and Pierce Western Blotting Substrate Plus (Thermo Fisher Scientific). To quantify the phosphorylation level of SARM1 and the protein level of NF-L and TH, the individual band images of proteins were scanned and analysed using ImageJ software. Then, their intensities were normalized against total SARM1 or β-Actin as an internal control.

### Immunohistochemistry

To optically detect dopaminergic neurons of mouse brain, we performed immunostaining of TH in the SNpc. Brain slices were blocked with 1% normal goat serum for 30 min and incubated with a mouse anti-TH monoclonal antibody (Santa Cruz Biotechnology, cat# sc-25,269, dilution 1:100 in 0.2% PBST) for 18 hr at 4 °C. After being washed in 0.2% PBST, the samples were incubated with Alexa Fluor 594 goat anti-mouse IgG antibody (Thermo Fisher Scientific, dilution 1:100 in 0.2% PBST) for 2 hr at RT. After being washed in 0.2% PBST, the samples were mounted using VECTASHIELD Mounting Medium with DAPI (Vector Laboratories). The specimens were observed using a fluorescence microscope (model BZ-X700; Keyence, Osaka, Japan). The relative density of TH-positive signals in the SNpc was measured quantitatively using ImageJ software.

### Cell viability assay

The CellTiter-Glo assay (Promega Biosciences, Madison, WI) was used to analyse cell viability. According to the manufacturer’s instructions, cells were incubated with CellTiter-Glo detection reagent for 10 min. Luminescence was observed using a Fluoroskan Ascent FL microplate fluorometer (Thermo Fisher Scientific). Cell viability was calculated with the control group at 100%.

### Quantitative cell imaging

To optically observe cell morphologies, we performed immunostaining of NF-L and MAP2 in neurons. At the end of treatment, the same volume of 4% PFA to medium was added into each well of plates to fix cells for 10 min at room temperature. After removal of the solution, the cells were fixed again by 4% PFA for 20 min. After being washed with PBS, the cells were permeabilized with 0.1% TritonX-100 in PBS for 20 min, then were incubated with a blocking buffer (10% skim milk in 0.1% PBST) for 20 min. Next, the cells were incubated with a mouse anti-NF-L monoclonal antibody (Santa Cruz Biotechnology, cat# c-20,012, dilution 1:200 in the blocking buffer) and a rabbit anti-MAP2 antibody (CST, cat# 8707, dilution 1:200 in the blocking buffer) for 18 hr at 4 °C. After being washed in PBS, the cells were incubated with Alexa 488 goat anti-mouse IgG antibody (Thermo Fisher Scientific, dilution 1:200 in the blocking buffer) and Alexa Fluor 594 goat anti-rabbit IgG antibody (Thermo Fisher Scientific, dilution 1:200 in the blocking buffer) for 2 hr at RT. After being washed in PBS, the cells were incubated with Hoechst 33342 (Thermo Fisher Scientific, dilution 1:2000 in the blocking buffer) for 20 min. For quantification of neurofilament integrity, the NF-L immunoreactivity was analysed by an In Cell Analyzer 2000 (Cytiva). Fluorescence images were acquired at the indicated time points using the In Cell Analyzer 2000 20× objective and were analysed using In Cell Analyzer 1000 Workstation software (Cytiva). Then, NF-L fluorescence intensities were normalized against nuclear numbers.

### ROS assay

CM-H_2_DCFDA (Thermo Fisher Scientific), a ROS indicator, was used to visualize intracellular ROS levels. Cells were incubated with 5 μM CM-H_2_DCFDA at 37 °C for 30 min. Then, the CM-H_2_DCFDA signal was observed using the fluorescent microscope (model BZ-X700; Keyence).

### Rhod 2 staining

Rhod 2-AM (Dojindo, Mashiki, Japan), a Ca^2+^ indicator, was used to visualize intracellular Ca^2+^ levels. Cells were incubated with 5 μM Rhod 2-AM in a loading medium (20 mM HEPES (pH 7.4), 115 mM NaCl, 5.4 mM KCl, 0.8 mM MgCl_2_, 1.8 mM CaCl_2_ and 13.8 mM glucose) at 37 °C for 30 min. After washing with PBS, the Rhod 2 signal was observed using the fluorescent microscope.

### Real-time qPCR

Total RNA was prepared using an SV Total RNA Isolation System (Promega Biosciences). First-strand cDNA synthesis was performed with total RNA using a SuperScript III First-Strand Synthesis System for RT-PCR (Thermo Fisher Scientific). Synthesized cDNA was used for the PCR analysis with *Power*SYBR Green PCR Master Mix (Thermo Fisher Scientific) and primers targeting the following gene transcripts: *NF-L* (F: CATCAGCGCTATGCAGGACA, R: TCACGTTGAGGAGGTCTTGG), *NF-M* (F: GAGCATCGAGCTAGAGTCGG, R: CTGGATGGTGTCCTGGTAGC) and glyceraldehyde 3-phosphate dehydrogenase (*GAPDH*) (F: GTCTCCTCTGACTTCAACAGCG, R: ACCACCCTGTTGCTGTAGCCAA). Their relative expression levels were calculated using the ΔCt method, normalized against *GAPDH* as an internal control and analysed using StepOnePlus software (Thermo Fisher Scientific).

### Statistical analysis

Before the statistical analyses, each experiment was repeated three times. The results are expressed as the mean ± standard deviation (SD). All statistical analyses were performed with EZR (Saitama Medical Center, Jichi Medical University, Saitama, Japan), which is a graphical user interface for R (The R Foundation for Statistical Computing, Vienna, Austria) *(**28**)*. A one-way analysis of variance (ANOVA) and a two-way ANOVA were used for comparison. If the ANOVA showed a significant difference, Tukey’s test was used as a post hoc test. *P*-values < 0.05 were considered statistically significant.

## Results

### Neurons derived from the PARK2 patient’s iPSCs were more vulnerable to rotenone-induced stress than those derived from the healthy donor iPSCs

To investigate the involvement of SARM1 phosphorylation and axonal degeneration in PD, we conducted a comparison study using normal (control) neurons and PD neurons in cultures. The neurons were prepared by a neuronal differentiation procedure for 6 days using neural stem cells (NSCs) originating from iPSCs that were derived from two healthy donors (201B7 and 585A1) and a familial PD patient with a homozygous deletion of *PARK2* exons (FPD2) *(**26**)*.

**Fig. 1:**
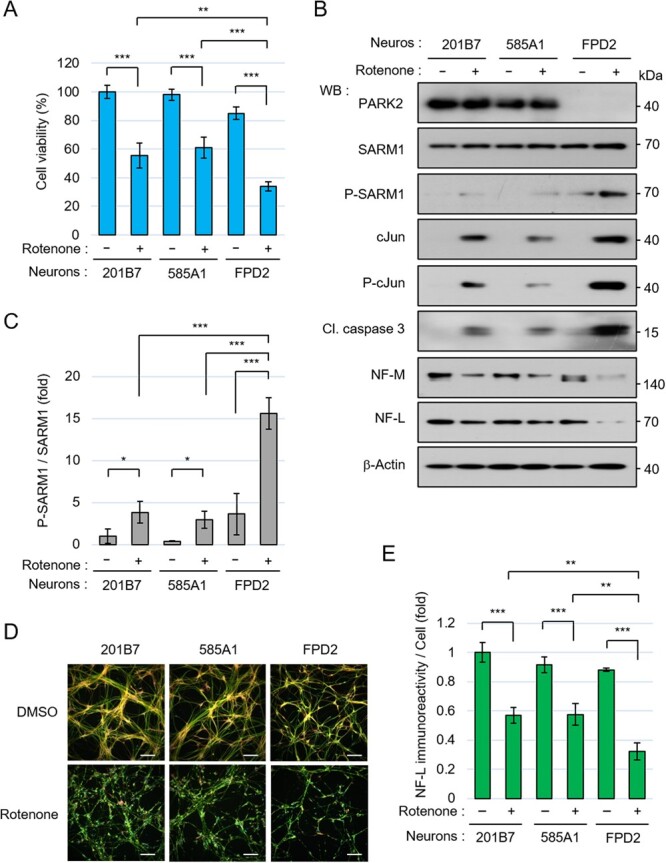
Compared to the control healthy neurons, the FPD2 neurons were more vulnerable to rotenone-induced stress. (A–E) Control 201B7-NSCs, Control 585A1-NSCs, and FPD2 NSCs were seeded at a density of 2.2 × 10 ^5^ cells/cm^2^ and differentiated into neurons for 6 days, and then were treated with 0.1% DMSO or 10 μM rotenone for 24 hr. (A) Cell viability was measured by the CellTiter-Glo assay. (B) Cell lysates of control and FPD2 neurons were analysed by western blotting with the indicated antibodies. (C) Quantification of the band intensity of phosphorylated SARM1. The level of phosphorylated SARM1 was normalized by total SARM1 protein. The data are expressed in fold changes using the DMSO-treated 201B7 neurons as control. (D) Immunostaining of NF-L, MAP2, and nucleus at 24 hr after treatment with DMSO or 10 μM rotenone. Scale bar: 100 μm. (E) Quantification of NF-L immunoreactivity. The neurofilament integrity was quantified by comparing the NF-L immunoreactivity with and without the addition of rotenone. The NF-L fluorescence intensities were normalized against nuclear numbers. The data are expressed in fold changes using the DMSO-treated 201B7 neurons as control. Error bars: mean ± SD of three independent experiments. ^*^P < 0.05, ^**^P < 0.01, ^***^P < 0.001.

When rotenone was added to the culture systems after neural differentiation, the viability of the FPD2 neurons was lower than that of the control neurons (Fig. 1A). We examined PD-relevant cellular molecules of our interest by western blotting. In the FPD2 neurons, PARK2 protein was not detected due to a homozygous deletion of *PARK2* exons (Fig. 1B). The phosphorylation level of SARM1 at Ser-548 induced by rotenone was higher in the FPD2 neurons compared to the control neurons (Fig. 1B and 1C). We had identified a stress kinase JNK that phosphorylates SARM1 at Ser-548 *(**25**)*. Since the phosphorylation level of a transcription factor cJun (another renowned substrate of JNK) was also increased by rotenone, it is reasonable to assume that the upstream kinase JNK is in an active state in FPD2 neurons in response to rotenone-induced stress. The total level of cJun was also increased by rotenone treatment (Fig. 1B), conceivably attributing to a transcriptional induction of it, which is self-regulated forward by its translated active product with the stress-mediated phosphorylation modification *(**29**)*. The plethora of events in FPD2 neurons observed in the WB image (Fig. 1B), such as mal-increase in phosphorylation of SARM1 and cJun in response to the stressor rotenone, probably attributed to compromised dysfunctional mitochondria stacked one after another in the FPD2 neurons. PARK2 protein owns a vital task to lead mitophagy to exclude dysfunctional mitochondria that will actively produce ROS. Within the expectation, ROS production resulted in higher levels in the FPD2 neurons than those in the control neurons, even under the standard non-stimulus condition and was much more increased in the FPD2 neurons by the treatment with rotenone (Fig. S1A). The mitochondrial ROS production enhances JNK, whose active phosphorylation modification is growing through the activation of JNK-upstream ASK1 and, inversely, the inactivation of JNK phosphatase *(**30**)*. The results therefore suggest that an irregularly regulatied ROS generation in a sustainable way from the stress-sensitive fragile mitochondria is one of the major causes of JNK activation and subsequent phosphorylation of SARM1 and cJun in the FPD2 neurons.

Regarding molecular events, the level of the apoptotic marker cleaved-caspase 3 and the degradation of the axon components NF-M and NF-L were both increased in FPD2 neurons compared to the control neurons (Fig. 1B). The mRNA levels of NF-M and NF-L did not change in all neurons regardless of the rotenone treatment (Fig. S1B and S1C). To learn the intracellular state of fibrous architecture composed of neurofilament, we quantified the immunostained-NF-L density in cells treated and not treated with rotenone and compared them; the rotenone-induced decrease in the NF-L immunoreactivity of the FPD2 neurons was more significant than that in the control neurons (Fig. 1D [image] and Fig. 1E [quantified data]). These results indicate that compared to the healthy control neurons, the FPD2 neurons were more vulnerable to rotenone-induced stress and had higher levels of SARM1 phosphorylation.

### Evaluation of the disease-relevant phenotypes of PARK2 in neurons derived from an isogenic control line

We next generated PARK2-knockdown NSC lines from healthy donor NSCs (201B7 NSCs) by using a CRISPR-Cas9-mediated gene editing system to determine whether the disease-relevant phenotypes observed in the FPD2 neurons would also be observed in cells of the same genetic background. Although we tried to establish PARK2-kockout NSC clones by single-cell cloning, the NSCs were unfortunately not able to survive under the single-cell culture conditions. We therefore used two bulk cell lines under the PARK2-knockdowned-mix cell culture conditions (cell subline names: PARK2-KD No.1 and PARK2-KD No. 2).

**Fig. 2:**
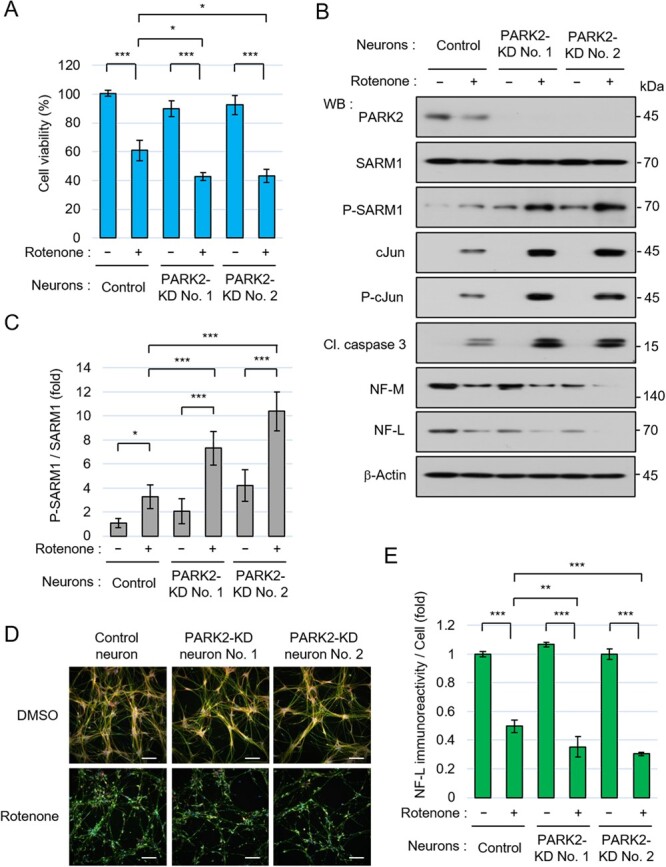
PARK2-knockdown (KD) neurons showed susceptibility to rotenone-induced stress. (A-E) PARK2-KD NSCs were produced by transfection of gRNAs targeting PARK2, the 1B-transposase plasmid, and the 1B-puromycin resistance gene plasmid to 201B7 NSCs and were selected by puromycin. Control NSCs were established by transfection the 1B-transposase plasmid and the 1B-puromycin resistance gene plasmid without gRNAs. Control and PARK2-KD (No. 1 and No. 2) NSCs were seeded at a density of 2.2 × 10 ^5^ cells/cm^2^ and differentiated into neurons for 6 days, and then treated with 0.1% DMSO or 10 μM rotenone for 24 hr. (A) Cell viability was measured by the CellTiter-Glo assay. (B) Western blot analysis of control and PARK2-KD neurons. The changes of cellular molecules 24 hr after the addition of DMSO or 10 μM rotenone were analysed. (C) Quantification of the band intensity of phosphorylated SARM1. The level of phosphorylated SARM1 was normalized by total SARM1 protein. The data are expressed in fold changes using the DMSO-treated control neurons as control. (D) Immunostaining of NF-L, MAP2, and nucleus at 24 hr after treatment with DMSO or 10 μM rotenone. Scale bar: 100 μm. (E) Quantification of NF-L immunoreactivity. The NF-L fluorescence intensities were normalized against nuclear numbers. The data are expressed in fold changes using the DMSO-treated control neurons as control. Error bars: mean ± SD of three independent experiments. ^*^P < 0.05, ^**^P < 0.01, ^***^P < 0.001.

The expression levels of PARK2 in their differentiated neurons were lower than those in the control neurons (Fig. 2B). The PARK2-KD neurons showed modestly increased susceptibility to the rotenone-induced stress (Fig. 2A). The levels of P-SARM1, P-cJun, and cleaved-caspase 3 were higher in the PARK2-KD neurons than those in the control neurons (Fig. 2B and 2C). The degradation rates of the axon components NF-M and NF-L were slightly increased in the rotenone-treated PARK2-KD neurons compared to those of the control neurons (Fig. 2B). The rotenone-induced decrease in the neurofilament density of the PARK2-KD neurons was greater than that in the control neurons (Fig. 2D and 2E). These results suggest that PARK2 is involved in rotenone-induced cell death and neurofilament degradation.

### JNK inhibitors suppressed rotenone-induced neurodegeneration

**Fig. 3:**
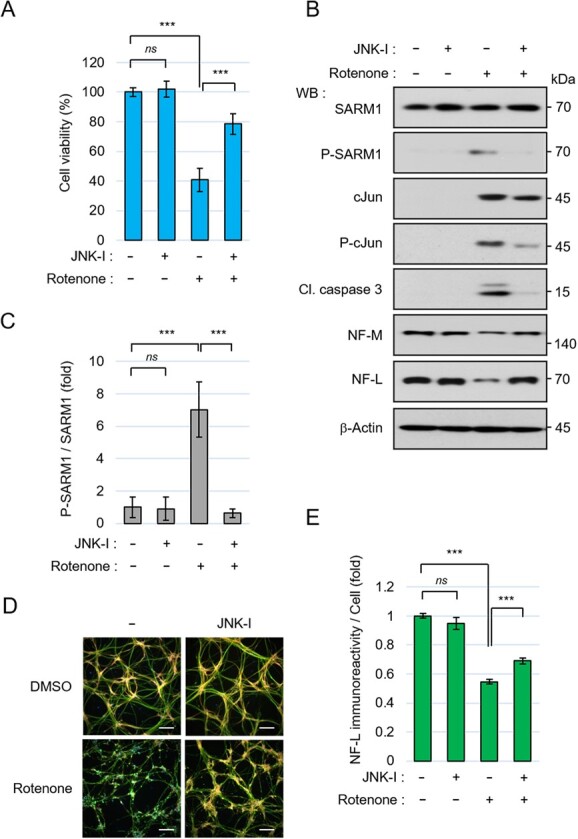
The JNK pathway is involved in rotenone toxicity. (A-E) 201B7 NSCs were seeded at a density of 1.5 × 10 ^5^ cells/cm^2^ and differentiated into neurons for 6 days, and then pretreated with 0.1% DMSO or 10 μM JNK inhibitor VIII for 30 min, then treated with 0.1% DMSO or 10 μM rotenone for 24 hr. (A) Cell viability was measured by the CellTiter-Glo assay. (B) Cell lysates treated with JNK inhibitor VIII and rotenone were analysed by western blotting with the indicated antibodies. (C) Quantification of the band intensity of phosphorylated SARM1. The level of phosphorylated SARM1 was normalized by total SARM1 protein. The data are expressed in fold changes using the DMSO-treated neurons as control. (D) Immunostaining of NF-L, MAP2, and nucleus at 24 hr after treatment with DMSO or 10 μM rotenone. Scale bar: 100 μm. (E) Quantification of NF-L immunoreactivity. The NF-L fluorescence intensities were normalized against nuclear numbers. The data are expressed in fold changes using the DMSO-treated neurons as control. Error bars: mean ± SD of three independent experiments. *ns*: not significant, ^***^P < 0.001.

As indicated in Fig. 1, the rotenone-induced cell death and neurofilament degradation may be associated with JNK activation. To verify whether JNK exerted these events, we tried to suppress JNK using chemical-based JNK inhibitors. The inhibitor used, JNK inhibitor VIII, effectively suppressed rotenone-induced cell death (Fig. 3A). Another JNK inhibitor, SP600125, also suppressed rotenone-induced cell death (Fig. S2A). These inhibitors caused reductions in the phosphorylation levels of the JNK substrates, SARM1 and cJun, followed by the suppression of caspase 3 cleavage and NF-L and NF-M degradation in response to rotenone stimulation (Fig. 3B, 3C, S2B and S2C). In addition, the attenuation of rotenone-induced neurofilament degradation in the presence of JNK inhibitors was confirmed by the quantification analysis of the NF-L immunoreactivity (Fig. 3D, 3E, S2D and S2E). These results indicate that the JNK signal is involved in rotenone-induced cell death and neurofilament degradation.

### SARM1 knockdown attenuated rotenone-induced neurodegeneration

**Fig. 4:**
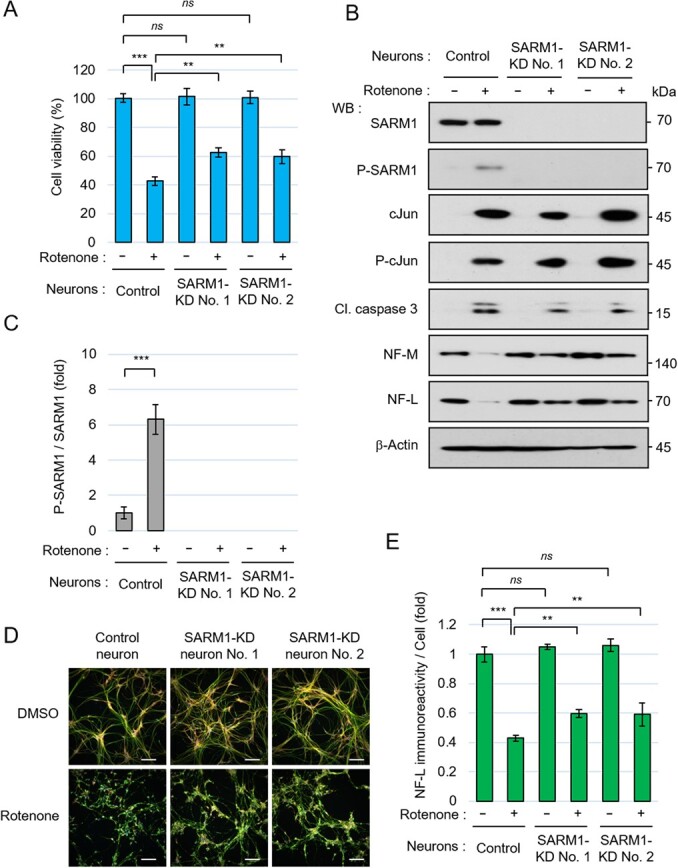
SARM1-knockdown (KD) neurons were resistant to rotenone toxicity. (A-E) SARM1-KD NSCs were produced by transfection of gRNAs targeting SARM1, the 1B-transposase plasmid, and the 1B-puromycin resistance gene plasmid to 201B7 NSCs and were selected by puromycin. Control NSCs were established by transfection of the 1B-transposase plasmid and the 1B-puromycin resistance gene plasmid without gRNAs. Control and SARM1-KD (No. 1 and No. 2) NSCs were seeded at a density of 1.5 × 10 ^5^ cells/cm^2^ and differentiated into neurons for 6 days, and then treated with 0.1% DMSO or 10 μM rotenone for 24 hr. (A) Cell viability was measured by the CellTiter-Glo assay. (B) Western blot analysis of control and SARM1-KD neurons. The changes of cellular molecules 24 hr after the addition of DMSO or 10 μM rotenone were analysed. (C) Quantification of the band intensity of phosphorylated SARM1. The level of phosphorylated SARM1 was normalized by total SARM1 protein. The data are expressed in fold changes using the DMSO-treated control neurons as control. (D) Immunostaining of NF-L, MAP2, and nucleus at 24 hr after treatment with DMSO or 10 μM rotenone. Scale bar: 100 μm. (E) Quantification of NF-L immunoreactivity. The NF-L fluorescence intensities were normalized against nuclear numbers. The data are expressed in fold changes using the DMSO-treated control neurons as a control. Error bars: mean ± SD of three independent experiments. *ns*: not significant, ^**^P < 0.01, ^***^P < 0.001.

We next turned our attention to SARM1 since it is a phosphorylation target of activated JNK and is now one of the notable molecules involved in neurodegeneration. To analyse the direct effect of SARM1 on rotenone-induced neurodegeneration, we generated SARM1-knockdown NSCs from healthy donor NSCs (201B7 NSCs) using a CRISPR-Cas9-mediated gene editing system. We used two bulk cell lines (mixed cell populations) of SARM1 knockdown (cell subline names: SARM1-KD No. 1 and SARM1-KD No. 2), and rotenone-induced cell death was modestly alleviated in the SARM1-KD neurons (Fig. 4A). We confirmed that although there was a clear presence of SARM1 bands and a phosphorylated band at the rotenone-treated side in the control neurons, there was almost no SARM1 protein or its phosphorylated type in the established SARM1-KD neurons (Fig. 4B and 4C). At that time, we observed that the level of phosphorylation of cJun induced by rotenone in the SARM1-KD neurons was almost the same as that in the control neurons (Fig. 4B), indicating that JNK is properly activated even under the SARM1-knockdown condition. In agreement with the results shown in Fig. 4A, the rotenone-induced increase in the level of cleaved caspase 3 was modestly decreased when SARM1 was knocked down (Fig. 4B). The rotenone-induced cell death was further suppressed in SARM1-KD neurons under the presence of a JNK inhibitor (Fig. S3A).

The degradation of the axon components NF-M and NF-L induced by rotenone was reduced in the SARM1-KD neurons (Fig. 4B). We further confirmed that the rotenone-induced decrease in the neurofilament density of the SARM1-KD neurons after rotenone addition was alleviated compared to the control neurons (Fig. 4D and 4E). These results indicated that SARM1 knockdown attenuated the rotenone-induced harmful effects on neurons, such as cell death and neurofilament degradation.

### SARM1 phosphorylation at Ser548 augmented the effect of rotenone

**Fig. 5:**
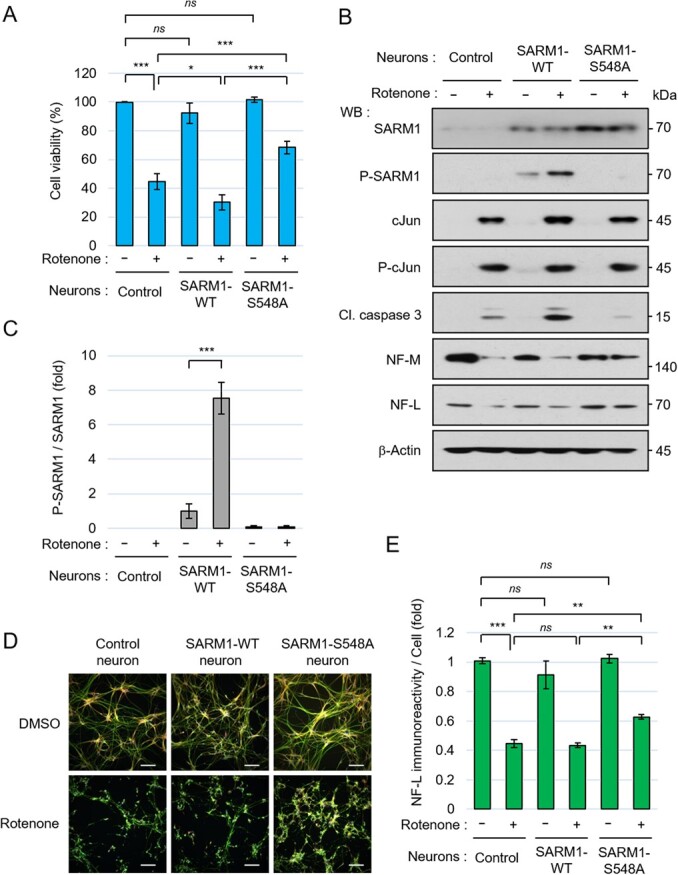
SARM1 phosphorylation at Ser-548 is involved in rotenone-induced cell death and neurofilament degradation. (A-E) To establish the gene-engineered NSCs that overexpress SARM1s, 201B7 NSCs were transfected with a set of plasmids, including the 1B-SARM1-WT or S548A plasmid, the 1B-transposase plasmid, and the 1B-puromycin resistance gene plasmid, and were selected by puromycin. The 1B-empty-gene plasmid was used instead of the 1B-SARM1 plasmid to obtain the control NSCs. Control, SARM1-WT-overexpressing, and SARM1-S548A-overexpressing NSCs were seeded at a density of 1.5 × 10 ^5^ cells/cm^2^ and differentiated into neurons for 6 days, and then treated with 0.1% DMSO or 10 μM rotenone for 24 hr. (A) Cell viability was measured by the CellTiter-Glo assay. (B) Western blot analysis of control, SARM1-WT-overexpressing, and SARM1-S548A-overexpressing neurons. The changes of cellular molecules 24 hr after the addition of DMSO or 10 μM rotenone were analysed. (C) Quantification of the band intensity of phosphorylated SARM1. The level of phosphorylated SARM1 was normalized by total SARM1 protein. The data are expressed in fold changes using the DMSO-treated SARM1-WT neurons as control. (D) Immunostaining of NF-L, MAP2, and nucleus at 24 hr after treatment with DMSO or 10 μM rotenone. Scale bar: 100 μm. (E) Quantification of NF-L immunoreactivity. The NF-L fluorescence intensities were normalized against nuclear numbers. The data are expressed in fold changes using the DMSO-treated control neurons as control. Error bars: mean ± SD of three independent experiments. *ns*: not significant, ^*^P < 0.05, ^**^P < 0.01, ^***^P < 0.001.

To further investigate the role of SARM1 phosphorylation, we established stable cell lines of NSCs that overexpress either wild-type SARM1 (WT) or SARM1 mutant in which the phosphorylation site Ser-548 was replaced by Ala (S548A) from 201B7 NSCs. The established NSC lines overexpressing SARM1-WT or S548A were differentiated into neurons and exposed to rotenone. The rotenone-induced cell death rate was higher in the SARM1-WT neurons than in the control neurons. On the other hand, SARM1-S548A expression worked against the cytotoxic effect of rotenone (Fig. 5A). The rotenone-induced cell death was further suppressed in SARM1-S548A neurons when combined with a JNK inhibitor for the treatment of the cells (Fig. S3B), likewise in the case of the similar treatment of SARM1-KD neurons (Fig. S3A). The phosphorylation of overexpressed SARM1-WT was increased by exposure to rotenone (Fig. 5B and 5C).

We also confirmed that there was no appreciable difference in the cJun phosphorylation level among the three lines of cells with rotenone treatment. In addition, in a comparison of the three lines of cells with rotenone treatment, the level of cleaved caspase 3 induced by rotenone was highest in the SARM1-WT-expressed neurons and lowest in the non-phosphorylatable S548A-expressed neurons (Fig. 5B). The detection of cleaved caspase 3 at the lowest level in the rotenone-treated SARM1-S548A neurons was also associated with suppression of the degradation of NF-M and NF-L and neurofilament density (Fig. 5B, 5D and 5E). These results indicate that SARM1 phosphorylation at Ser548 modulates rotenone-induced cell death and neurofilament degradation.

### Involvement of Ca^2+^ signal in rotenone-induced neurodegeneration

It has been reported that SARM1 metabolizes NAD^+^ and produces cyclic ADP-ribose (cADPR) *(**13**,**24**)*, which acts as a signal messenger to increase intracellular the Ca^2+^ concentration *(**31*–*33**)*. We confirmed that the overexpression of SARM1-WT caused an increase in the free Ca^2+^ level in transfected HEK293T cells, and the co-overexpression of SARM1-WT and JNK1 further enhanced Ca^2+^ production capacity than the overexpression of SARM1-WT alone (Fig. S3C). We also found that the overexpression of non-phosphorylatable SARM1-S548A induced lower levels of free Ca^2+^ than SARM1-WT, and the overexpression of a TIR-deletion mutant of SARM1 (ΔTIR, deletion of 551–724 amino acids) that lacks NAD^+^ cleavage ability had no effect on the intracellular mobilization of free Ca^2+^ (Fig. S3C).

**Fig. 6:**
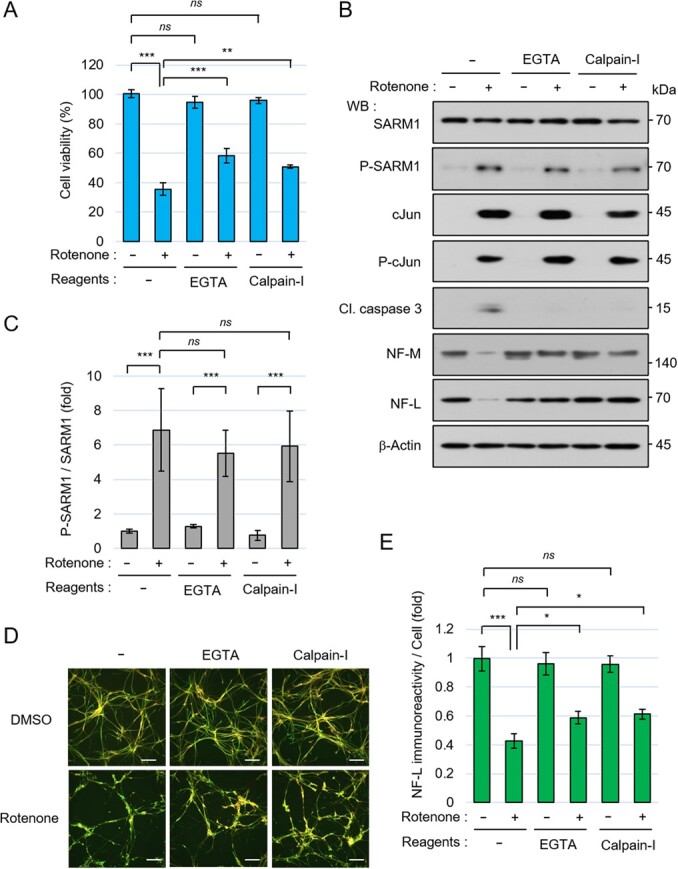
Ca ^2+^ signal is involved in rotenone toxicity. (A-E) 201B7 NSCs were seeded at a density of 1.5 × 10^5^ cells/cm^2^ and differentiated into neurons for 6 days, and then pretreated with 0.1% DMSO, 2 mM EGTA, or 80 μM calpain inhibitor III for 30 min; the neurons were then treated with 0.1% DMSO or 10 μM rotenone for 24 hr. (A) Cell viability was measured by the CellTiter-Glo assay. (B) Cell lysates treated with the indicated inhibitors and rotenone were analysed by western blotting. (C) Quantification of the band intensity of phosphorylated SARM1. The level of phosphorylated SARM1 was normalized by total SARM1 protein. The data are expressed in fold changes using the DMSO-treated neurons as a control. (D) Immunostaining of NF-L, MAP2, and nucleus at 24 hr after treatment with DMSO or 10 μM rotenone. Scale bar: 100 μm. (E) Quantification of NF-L immunoreactivity. The NF-L fluorescence intensities were normalized against nuclear numbers. The data are expressed in fold changes using the DMSO-treated neurons as a control. Error bars: mean ± SD of three independent experiments. *ns*: not significant, ^*^P < 0.05, ^**^P < 0.01, ^***^P < 0.001.

We used the Ca^2+^ chelator EGTA to investigate the involvement of the Ca^2+^ signal in rotenone-induced cell death and neurofilament degradation. Since an intracellular increase in Ca^2+^ to a high level induces axonal degeneration through the activation of calpain *(**34**,**35**)*, a calpain inhibitor was used in additional experiments. By this inhibitory approach, we found that EGTA and a calpain inhibitor suppressed rotenone-induced cell death (Fig. 6A). The inhibitory function of cell death was also confirmed by the results of western blotting showing that the cleavage of caspase 3 was suppressed by EGTA and the calpain inhibitor (Fig. 6B).

Under the same conditions, we observed that the levels of the rotenone-induced phosphorylation of SARM1 and cJun were not dramatically altered by treatment with EGTA or a calpain inhibitor compared to the levels in rotenone-treated control cells (Fig. 6B and 6C). The rotenone-induced degradation of the axonal components NF-M and NF-L was suppressed by treatment with EGTA and the treatment with a calpain inhibitor (Fig. 6B). The inhibition of neurofilament degradation was also confirmed by our quantification of the NF-L immunoreactivity (Fig. 6D and 6E). These results suggest that the Ca^2+^ signal works downstream of SARM1 and is involved in rotenone-induced cell death and neurofilament degradation.

### SARM1 was phosphorylated in the midbrain of rotenone-treated mice

**Fig. 7:**
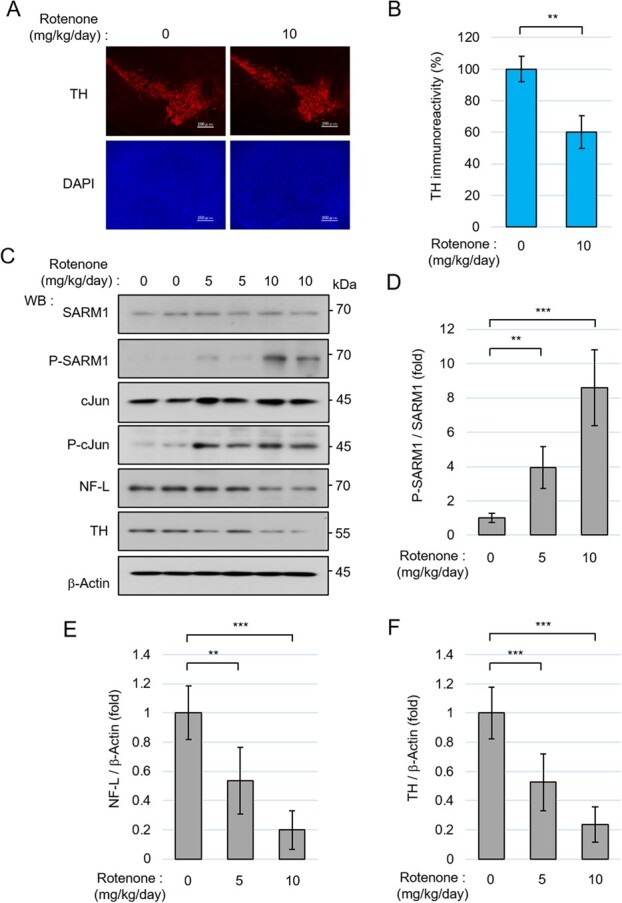
SARM1 is phosphorylated in the midbrain of mice treated with rotenone. (A) Representative images of immunostaining for tyrosine hydroxylase (TH) and nucleus (DAPI) in the substantia nigra pars compacta (SNpc) of mice 4 weeks after vehicle or rotenone treatment. Scale bar: 200 μm. (B) Changes in the signal intensity of TH immunoreactivity in the SNpc. (C) Lysates from the midbrain containing SNpc 4 weeks after vehicle or rotenone treatment were analysed by western blotting with the indicated antibodies. (D) Quantification of the band intensity of phosphorylated SARM1 in the midbrain. The level of phosphorylated SARM1 was normalized by total SARM1 protein. (E and F) Quantification of the band intensity of NF-L and TH in the midbrain. The levels of protein were normalized by β-Actin. The data are expressed in fold changes using the no-treated group as a control. The data are mean ± SD (*n* = 3 mice per group). ^**^P < 0.01, ^***^P < 0.001.

To determine the pathological significance of SARM1 phosphorylation at Ser-548 *in vivo*, we used a rotenone-treated PD mouse model in which rotenone was manipulated to be gradually released with diffusion in the subcutaneous area over a period of 28 days. Dopaminergic neurons are lost in this PD mouse model *(**36**,**37**)*. Herein, the loss of dopaminergic neurons was confirmed by the administration of rotenone at 10 mg/kg/day using immunostaining for tyrosine hydroxylase (TH), a dopaminergic neuron marker (Fig. 7A). In our procedure at that rotenone dose, the brains of the model mice showed a 40% decrease in TH immunoreactivity in SNpc (Fig. 7B).

To examine the expression and phosphorylation status of SARM1 and relevant molecules, we surgically resected individual midbrain parts containing the SNpc from model mice loaded with 0, 5, or 10 mg/kg/day of rotenone, and the specimens were analysed by western blotting. The *in vivo* administration of rotenone increased the SARM1 phosphorylation in the midbrain (Fig. 7C and 7D). The phosphorylation of cJun was also increased in the midbrain, suggesting the presence of the active form of JNK. Concomitant with the increases in the phosphorylation of SARM1, decreases in NF-L and TH were also observed (Fig. 7C, 7E and 7F). These results suggest that rotenone induces SARM1 phosphorylation *in vivo* and induces dopaminergic neuron death accompanied by axonal degradation.

## Discussion

The findings obtained in this study revealed the pathological changes of SARM1 phosphorylation at Ser-548 in neuronal cells in an *in vitro* culture system as well as in an *in vivo* mouse model of PD. Exposure to rotenone, which induces PD-like symptoms, induced SARM1 phosphorylation, which is induced by JNK activation, and the phosphorylated SARM1 was involved in neurofilament degradation and cell death. Our results further demonstrated that the SARM1-derived Ca^2+^ signal plays an important role in rotenone-induced neurodegeneration through the activation of Ca^2+^-sensitive calpain.

Our *in vitro* studies focusing on the cell phenotype in iPSC-derived neuronal cells from healthy donors and a PARK2 patient showed that the viability of the FPD2 neurons was lower than that of the control neurons and that the lower viability of the FPD2 neurons was associated with a high level of SARM1 phosphorylation (Fig. 1). Since FPD2 neurons have a mutation in *PARK2* gene, abnormal mitochondria may accumulate in the cells due to a defect of mitophagy. Imaizumi et al. reported that FPD2 neurons have abnormal mitochondria with irregular shapes that are linked to increased oxidative stress *(**26**)*. We also confirmed that the ROS level was highly elevated in the FPD2 neurons, which is distinct from those in control neurons (Fig. S1A). Since SARM1 activated by phosphorylation at Ser-548 causes an inhibition of mitochondrial respiration *(**25**)*, the activated form may directly affect mitochondria and change them to an abnormal type in FPD2 neurons.

To analyse the direct effect of PARK2 on rotenone-induced neurodegeneration, we conducted experiments using a CRISPR-Cas9-mediated gene editing system. Since NSCs were not able to survive under the setting of single-cell culture, we used bulk cell lines under PARK2-knockdown conditions (Fig. 2). Although the results in Fig. 2 showed the same tendency as those in Fig. 1, the results under PARK2-knockdown conditions were less clear than those under the PARK2-null conditions. The reasons for the above results may be due to the use of the heterogenous mixed population of the PARK2-KD cells that may have included diverse cells expressing different levels of *PARK2* gene because of incomplete genetic ablation; alternatively, the FPD2 neurons and PARK2-KD neurons may have had different genetic backgrounds. Further analyses using iPSCs from many donors and isogenic iPSCs lacking *PARK2* are necessary to address this question.

SARM1 metabolizes NAD^+^ and produces cADPR, which acts to increase the intracellular level of Ca^2+^. Tabata et al. reported that the intracellular Ca^2+^ level is elevated in neurons derived from PARK2 patients and that the inhibition of T-type Ca^2+^ channels suppress rotenone-induced neuronal cell death *(**38**)*. Similarly, our present experiment confirmed that a chelating procedure of intracellular Ca^2+^ using EGTA attenuates the rotenone-induced neuronal cell death and neurofilament degradation (Fig. 6). In light of the report that an increase of Ca^2+^ in cells leads to a sustained activation of the TAK1-JNK pathway *(**39**)*, it is reasonable to speculate that irregularly mobilized Ca^2+^ may trigger the formation of a signal loop (like JNK-SARM1-Ca^2+^-TAK1-JNK) in a sustained manner, contributing to the pathological progression of PD in PARK2 patients.

The effect of the non-phosphorylatable variant SARM1-S548A attracted our attention since the prepared neurons that overexpress SARM1-S548A in culture had less rotenone-induced cell death and neurofilament degradation compared to the healthy control neurons (Fig. 5). This suggests that the phosphorylation modification of SARM1 at Ser-548 affects neuronal cell survival. Since protein structural analyses have shown that SARM1 forms an oligomer *(**19**,**21**,**40**)*, we suspected that overexpressed SARM1-S548A integrates into the oligomer as a dominant negative effecter. Geisler et al. developed an adeno-associated virus vector expressing a SARM1 mutant that blocks endogenous SARM1 enzymatic activity, and they showed by using this vector that the developed SARM1 blocker effectively suppresses axonal degeneration *in vivo (**41**)*. SARM1-S548A mutant may have a similar inhibitory effect. In fact, Xue et al. reported that in cerebral ischemia/reperfusion injury model, the overexpression of SARM1-S548A delivered with the lentivirus vector reduced the infarct size, the rate of neuronal cell death, and neurobehavioural dysfunction *(**42**)*.

The details of the molecular machinery by which the phosphorylation of Ser-548 on SARM1 leads to the functional activation of SARM1 remain to be established. Some studies have shown that the NAD^+^ salvage pathway regulates SARM1 activity *(**13**,**19**,**24**)* and that nicotinamide mononucleotide adenylyltransferase 2 (NMNAT2), which is a NAD^+^ synthetic enzyme, lowers the NMN level and maintains the NAD^+^ level to suppress SARM1 activation *(**43*–*47**)*. It has also been shown that NMN and a cell-permeant mimetic of NMN induce SARM1 activation by enhancing the liberation of the TIR domain (the catalytic domain), which enables flexible motion of the TIR domain and leads to an active conformational change of SARM1 with its oligomerization via the liberated TIR-TIR interactions *(**19**,**24**,**48**)*. This attractive machinery will help to unravel the molecular mechanisms underlying the phosphorylation-triggered activation of SARM1. We are curious about whether the phosphorylation modification of SARM1 at Ser-548 caused by JNK orchestrates with NMN stimulation that may cooperatively accelerate the conformational change of SARM1 towards its active form. If that is the case, we need to clarify which process(es), i.e., the liberation of the TIR domain, TIR-TIR interactions, or the stabilization of the oligomerization, are accelerated by the phosphorylation.

Generally, proteins possessing a TIR domain are thought to be involved in the innate immune pathway. For example, TIR domains in Toll-like receptors (TLRs), interleukin-1 receptors (IL-1Rs), and their adaptor proteins (referred to as TIR adaptor proteins) are all involved in immune and inflammatory signalling *(**49**)*. Since SARM1 was identified as the fifth TIR adaptor protein, SARM1 was considered to play a role only in immune and inflammation signals, but SARM1 is also involved in axonal degeneration based on the NAD^+^ cleavage activity. We thus suspect that the NAD^+^ cleavage activity is a specific feature of the SARM1-TIR domain. However, intriguingly, recent studies have shown that this feature is not restricted to the TIR domain of SARM1, and that TIR domains of other proteins have NAD^+^ cleavage activity. The NAD^+^ cleavage activity of TIR domains has been detected in not only mammals but also plants and even archaea *(**20*–*22**)*. These findings suggest that TIR domain-induced NAD^+^ cleavage is a conserved function of TIR domains from higher to lower animals. Some TIR domains (including the TIR domain in SARM1) may therefore work together additively or synergistically for NAD^+^ cleavage in nerve and immune systems in response to several pathological settings related to not only degenerative diseases but also pathogen-related inflammatory diseases.

As mentioned above, the TIR domain also works as an adaptor for signal transduction, which has been studied in signal cascades of TLRs in immune and inflammation settings, in which TIR-TIR interactions between different TIR adaptors are important. Considering this function of TIR, SARM1-TIR may contribute to not only homo-interaction by itself but also hetero-interaction with other TIR-natured molecules, which may be induced by a JNK-mediated phosphorylation of SARM1 at Ser-548. It has been reported that SARM1 is involved in neuroinflammation and inflammasome activation *(**50**,**51**)* and that TLR4-mediated neuroinflammation and inflammasome activation contribute to the pathological process of PD *(**52**)*.

In summary, our present findings revealed that the phosphorylation of SARM1 plays an important role in rotenone-induced neurodegeneration. We hope that our results will help provide a foundation for establishing an effective treatment for PD.

## Data Availability

The data generated in this study and materials are available within the article and its supplementary data files.
